# A neuroanatomical and cognitive model of impaired social behaviour in frontotemporal dementia

**DOI:** 10.1093/brain/awae040

**Published:** 2024-02-09

**Authors:** Matthew A Rouse, Richard J Binney, Karalyn Patterson, James B Rowe, Matthew A Lambon Ralph

**Affiliations:** MRC Cognition and Brain Sciences Unit, University of Cambridge, Cambridge CB2 7EF, UK; Cognitive Neuroscience Institute, Department of Psychology, School of Human and Behavioural Sciences, Bangor University, Bangor LL57 2AS, UK; MRC Cognition and Brain Sciences Unit, University of Cambridge, Cambridge CB2 7EF, UK; Department of Clinical Neurosciences, University of Cambridge, Cambridge CB2 0SZ, UK; MRC Cognition and Brain Sciences Unit, University of Cambridge, Cambridge CB2 7EF, UK; Department of Clinical Neurosciences, University of Cambridge, Cambridge CB2 0SZ, UK; Department of Neurology, Cambridge University Hospitals NHS Foundation Trust, Cambridge CB2 0SZ, UK; MRC Cognition and Brain Sciences Unit, University of Cambridge, Cambridge CB2 7EF, UK

**Keywords:** frontotemporal dementia, semantic dementia, social-semantic knowledge, social control, anterior temporal lobe, orbitofrontal cortex

## Abstract

Impaired social cognition is a core deficit in frontotemporal dementia (FTD). It is most commonly associated with the behavioural-variant of FTD, with atrophy of the orbitofrontal and ventromedial prefrontal cortex. Social cognitive changes are also common in semantic dementia, with atrophy centred on the anterior temporal lobes. The impairment of social behaviour in FTD has typically been attributed to damage to the orbitofrontal cortex and/or temporal poles and/or the uncinate fasciculus that connects them. However, the relative contributions of each region are unresolved.

In this review, we present a unified neurocognitive model of controlled social behaviour that not only explains the observed impairment of social behaviours in FTD, but also assimilates both consistent and potentially contradictory findings from other patient groups, comparative neurology and normative cognitive neuroscience. We propose that impaired social behaviour results from damage to two cognitively- and anatomically-distinct components. The first component is social-semantic knowledge, a part of the general semantic-conceptual system supported by the anterior temporal lobes bilaterally. The second component is social control, supported by the orbitofrontal cortex, medial frontal cortex and ventrolateral frontal cortex, which interacts with social-semantic knowledge to guide and shape social behaviour.

## Teaser

Rouse *et al.* propose an integrative neurocognitive model of controlled social behaviour that can explain the observed impairment of social behaviours in frontotemporal dementia. The model can also account for findings from other patient groups, including individuals with semantic dementia, and from studies of the healthy brain.

## Introduction

Impaired social behaviour is a common manifestation of frontotemporal dementia (FTD). For example, people with FTD may make insensitive comments, show inappropriate levels of familiarity with strangers or disregard social norms and etiquette.^1^ Apathy and impulsivity are common exacerbating factors in abnormal social behaviour, with reduced engagement in social activities and disinhibited behaviours co-occurring in FTD.^2-4^ These behavioural disturbances in FTD can have a devastating impact; they cause significant burden and stress for family members and caregivers^5^ and predict care home admission.^6^

FTD is split into two main subtypes: behavioural-variant FTD (bvFTD) and primary progressive aphasias. The latter includes semantic dementia that encompasses semantic-variant primary progressive aphasia (svPPA) and its right-temporal homologue.^7-9^ In terms of the underlying focus of pathology, bvFTD predominantly affects the prefrontal cortex and is characterized by changes in behaviour and personality as well as a dysexecutive neuropsychological profile.^7^ Semantic dementia is associated with atrophy centred on the ventrolateral and polar aspects of the bilateral anterior temporal lobes (ATLs), coupled with degraded semantic knowledge across all types of concepts and observed in all verbal and nonverbal modalities.^8,10-13^ It is well-established that behavioural changes are found not only in bvFTD but are also common in semantic dementia. Large-scale studies find similar rates of behaviour change in both bvFTD and semantic dementia subtypes.^3,14-17^

Whilst the frontal and temporal lobes have been implicated in supporting socially appropriate and pro-social behaviours,^18,19^ the precise contributions of each region are not clear in either syndrome and in their common symptoms. This represents both an important gap in clinical knowledge and an unresolved theoretical issue, in part caused by the fact that key information is distributed across multiple disparate literatures on each FTD subtype, as well as findings from other patient groups, and from healthy participants.^20-23^

**Figure 1:**
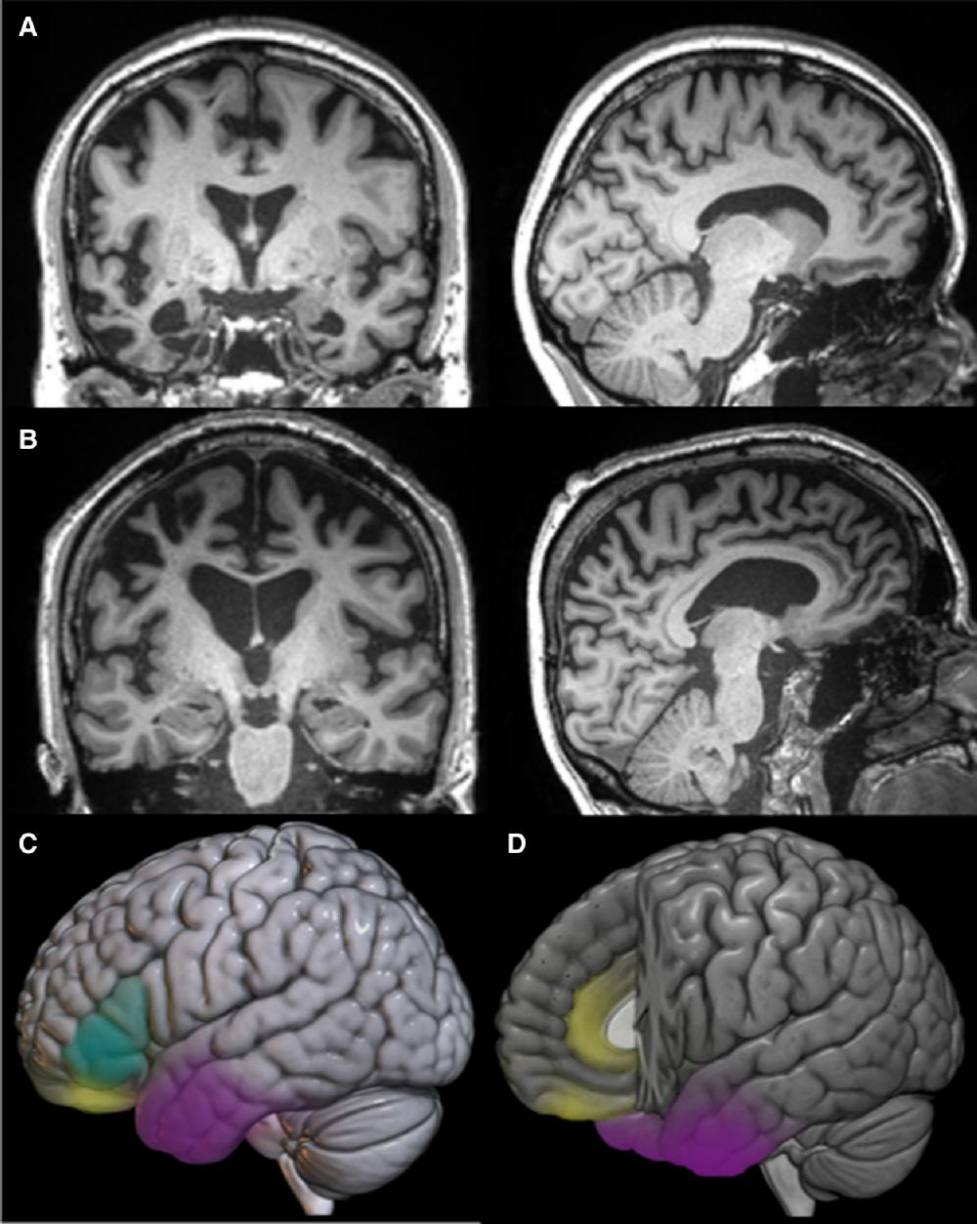
**The controlled social-semantic cognition model.** (**A**) Example coronal and sagittal MRI slices of a person with semantic dementia, displaying bilateral anterior temporal lobe atrophy. Images are shown in neurological convention (i.e. left = left, right = right). (**B**) Example coronal and sagittal MRI slices of a person with behavioural-variant frontotemporal dementia, showing prefrontal atrophy. Images are shown in neurological convention (i.e. left = left, right = right). (**C**) A neuroanatomical sketch of the key areas within the controlled social-semantic cognition model. Social-semantic knowledge is represented in the anterior temporal lobe (purple). Social control is supported by orbitofrontal cortex (yellow) as well as ventrolateral prefrontal cortex (cyan). (**D**) Additional neuroanatomical sketch, this time cut out to display the medial prefrontal areas important for social control.

In this review, we propose an integrative neurocognitive model: ‘controlled social-semantic cognition’ (CS-SC). The model provides a unified frontotemporal framework for social behaviours that accounts for the findings from bvFTD, semantic dementia and ATL-resected temporal lobe epilepsy (TLE) patients, drawing on comparative neurology and studies of the healthy brain. Specifically, the model proposes that impaired social behaviour can result from damage to two distinct albeit interactive components: (i) social-semantic ‘knowledge’, underpinned by the bilateral ATLs; and (ii) social ‘control’, including selection, evaluation, decision-making and inhibition supported by frontal cortical regions, particularly the orbitofrontal cortex (OFC), medial prefrontal cortex and lateral prefrontal cortex (Fig. 1C and D). The proposal that semantic representations interact with prefrontal control processes to guide social behaviour mirrors the broader theory of controlled semantic cognition.^21,24^

According to the CS-SC framework, impaired social behaviour in FTD may result from damage to either of these components (or both). A key hypothesis of the framework is that semantic dementia patients have impaired behaviour due predominantly to a degradation of social-semantic knowledge, whilst bvFTD patients have earlier and disproportionate deficits in the ability to control and regulate social-semantic knowledge effectively, to guide appropriate and adaptive social behaviours. In this review, we describe the CS-SC model. We take the two components in turn, and, for each, we review evidence from multiple clinical disorders, comparative neurology and healthy participants. We then consider how the model and associated findings relate to previous proposals for explaining some of the behavioural changes in FTD. We end by setting out some key issues for further research and clinical implications.

The ‘multiple-literatures’ approach adopted in this review is crucial for at least two key reasons. First, models and theories are most powerful when they go beyond an individual result and are able to explain findings from several patient groups and contrastive neuroscience methods—especially when those findings are potentially contradictory. Second, each method or clinical condition has its own intrinsic advantages but also limitations. By assimilating data, it is possible to mitigate method/study-group limitations and focus on the complementary strengths and insights proffered by the other data sources, thereby converging upon a unified, coherent framework. As examples from the current review: whilst the behavioural and semantic deficits are substantial and paradigmatic of FTD, their precise localization is hampered by the correlated atrophy across multiple brain regions in FTD. In contrast, ATL resection for TLE provides a selective lesion model of the ATLs individually and separately from other frontotemporal areas, although the patients’ chronic epilepsy raises a possibility that re-organization of function may have occurred. Functional neuroimaging techniques such as functional MRI (fMRI) allow localization of brain function simultaneously across multiple areas and at a much higher spatial resolution than lesion studies, but can only indicate correlations. Causal brain-behaviour relationships in healthy participants can be elucidated using transcranial magnetic stimulation; however, the transient behavioural changes induced are considerably subtler than those observed after brain lesions.

## The anterior temporal lobes and social-semantic knowledge

We use our conceptual knowledge of the world to support everyday verbal and non-verbal behaviours. This long-term database of the meaning of words, objects, people and behaviours is known as semantic memory or conceptual knowledge^25^ and is critical if one is to generate appropriate social behaviours across different scenarios and contexts. For example, when a grandparent hugs a child who is upset, there are several semantic details and potential ambiguities which must be resolved. The grandparent must correctly recognize from the multiple sensory inputs that the young human is her/his grandchild, as well as understanding the meaning of the sounds, signals and tears that the child is generating, plus the meaning of the context/situation. In turn, the adult must then use semantic knowledge of the social role of grandparent to generate an appropriate comforting behaviour.

Now imagine the possible consequences that could occur following the degradation of semantic memory/conceptual knowledge. Failing to recognize the meaning of the signals of emotional distress would result in a failure to exhibit the socially appropriate behaviour. Semantic degradation could also lead to an inability to distinguish between one’s relative and other unfamiliar persons (e.g. if the child were not this adult’s grandchild), resulting in an overgeneralization of semantic knowledge^26^ and thus to another inappropriate social behaviour: to hug a stranger’s child. Accordingly, semantic knowledge is critical and foundational for understanding and generating social behaviours.^27^ We propose that this knowledge primarily relies on the same cognitive processes and brain regions that support other forms of semantic memory; indeed, there is a wealth of evidence that the bilateral ATLs act as a core transmodal, transtemporal, pan-category hub for generalizable conceptual knowledge.^21,24,28,29^ Although we refer to social-semantic knowledge throughout the review, all aspects of conceptual knowledge play a critical role in supporting behaviour.

### Semantic dementia

People with semantic dementia display a gradual loss of understanding for words, objects, people, etc.^8,10,11^ This progressive semantic degradation occurs for all types of concepts, across all modalities and in both expressive and receptive tasks.^13,30^ Structural neuroimaging and PET (Fig. 1A) demonstrate that the degree of semantic impairment in semantic dementia is correlated with ATL volume loss and hypometabolism.^31-33^ These findings, together with formal computational models^34-36^ and other convergent data (see later subsections) support the proposal that the ATLs form a transmodal, transtemporal semantic hub.^24,28,34^ Through dynamic interactions with modality-specific ‘spokes’ distributed throughout the cortex, the ATL hub integrates multimodal information for each concept (transmodal) across time and contexts (transtemporal), resulting in the extraction of generalizable, coherent concepts.^26^ Another core feature of the hub and spoke model is that the bilateral ATLs form a functionally-singular hub, which has been demonstrated computationally to make the semantic system more robust to unilateral damage or perturbation.^37^

Semantic dementia patients can present with overlapping clinical symptoms to those observed in bvFTD,^38,39^ with equal rates of reported behaviour change across these FTD subtypes.^16,17^ However, relative to the core semantic deficit, much less is understood about social processing deficits in semantic dementia. During the early stages of disease, the distribution of ATL atrophy in this condition is often asymmetric (but see ‘Other important brain areas and connectivity’ section). Case studies have revealed that patients with predominantly right-sided atrophy initially present with prosopagnosia and this is followed by emergence of behaviour change and/or the classical generalized semantic impairment.^33,40-43^ Impairments in social cognitive processes such as empathy, theory of mind and social conceptual knowledge have been associated with right ATL atrophy or hypometabolism.^44-46^ This has led to proposals that: (i) the right ATL has a specialized role in social processing^19,41,47^; and (ii) cases with right (R) > left (L) temporal atrophy might represent a unique clinical syndrome (Box 1).^9,41,48^

Box 1 **The conundrum of right anterior temporal lobe atrophy and social behaviour**

Frontotemporal dementia (FTD) patients with predominantly right (R) > left (L) anterior temporal lobe (ATL) atrophy often present in clinic with behavioural disturbances alongside difficulties in recognizing familiar people. Indeed, the behavioural changes can be hard to distinguish from those found in behavioural-variant FTD.^38^ Although there are well-studied R > L cases that do not follow this pattern,^42^ this presentation of R > L patients is routinely observed in clinics.^40,49,50^ Formal group comparisons have confirmed behavioural changes as a core symptom in R > L cases, although, importantly, they are often also found in L > R cases who tend to have less disease burden overall (see main text).^9,41^

Efforts have been made to conceptualize R > L semantic dementia as a discrete clinical syndrome, motivated in part because the recent consensus criteria for semantic variant primary progressive aphasia (svPPA) do not include face recognition problems and behaviour change.^8^ This has led to several alternative proposals for diagnostic criteria and an appropriate clinical label.^9,41,48^ The syndrome has been called ‘right temporal variant of FTD’ with proposed core clinical features including prosopagnosia, memory deficits and behaviour change.^48^ In parallel, the term ‘semantic behavioural-variant FTD’ has been proposed with diagnostic criteria including a selective degradation of person-specific semantic knowledge and loss of empathy.^9^ The proposed underlying cognitive mechanism for these symptoms is a loss of social-semantic knowledge following right ATL atrophy.^9^ It has also recently been suggested that the clinical syndrome associated with R > L ATL atrophy may partially reflect reward disturbances and a shift of hedonic value away from other people and towards inanimate objects.^51^ It should be noted that both typical L > R semantic dementia (svPPA) and R > L semantic dementia are usually associated with the same underlying TDP-43 type C neuropathology.^52^ In addition, the clinical phenotypes converge over time^47,53^ as atrophy increases rapidly in the contralateral ATL.^54,55^ Therefore, rather than considering right semantic dementia as a distinct syndrome, it may be more appropriate to conceptualize semantic dementia as a continuous spectrum, with people with L > R or R > L ATL atrophy located at opposing endpoints.^33,51,56^

According to the controlled social-semantic cognition (CS-SC) framework, social-semantic knowledge alongside conceptual representations more generally is represented bilaterally across the ATLs. In other words, the behaviour deficits in R > L semantic dementia do not occur because the right ATL has a specialized role in social cognition/social conceptual knowledge. How then, can our framework explain why people with R > L ATL atrophy often present with impaired social behaviour? It is important to acknowledge that when formally assessed, L > R semantic dementia patients can display behavioural disturbances too,^41,53,57^ with a recent study reporting that social-semantic knowledge correlated with bilateral ATL atrophy.^58^ R > L semantic dementia cases often present to clinic at a later stage in their disease relative to L > R cases, with cases of early, mild R > L semantic dementia cases being much rarer than their left-sided counterparts.^33,59^ When directly compared, R > L semantic dementia cases not only have more overall temporal lobe atrophy than L > R semantic dementia^33,41^ but often have greater atrophy in other frontotemporal areas such as the orbitofrontal cortex and anterior cingulate cortex.^33,47^ In light of these additional correlated factors, there are then two possible causes of the increased behaviour change in R > L semantic dementia: (i) R > L semantic dementia cases have greater overall ATL volume loss, bilaterally, leading to greater degradation of semantic knowledge required for appropriate social behaviour; and/or (ii) R > L semantic dementia cases have greater concurrent prefrontal damage, leading to increased problems with social control; or perhaps, most likely, a combination of the two factors.

There are caveats in the interpretation of asymmetric semantic dementia patients, given that the disease is never isolated to one ATL. Although atrophy may be asymmetric in the initial stages, hypometabolism tends to be more symmetrical even early in the disease,^60^ and longitudinal studies show that atrophy advances even more rapidly in the contralateral hemisphere.^47,54,55,61^ Accordingly, from here, we will refer to the asymmetric-yet-bilateral cases as L > R and R > L. Direct comparisons between R > L and L > R patients are confounded by the fact that, by the time that people with semantic dementia come to medical attention, R > L patients often have more overall atrophy than L > R patients.^33^ Even when they are matched for temporal lobe atrophy, comparisons have revealed that R > L cases have more atrophy extending into the OFC, which may contribute to the patients’ increased behavioural changes.^33,55^

Formal assessment of L > R semantic dementia patients shows that social processing and behaviour disturbances are prominent too.^41,47,53^ Therefore, the contributions of the ATLs to social behaviour appear to relate to atrophy of the left and/or right ATLs (and/or co-occurring atrophy within the frontotemporal distribution). Accordingly, the fact that behavioural disturbances are often noted in R > L semantic dementia cases might need to be considered in the context of multiple correlated factors beyond the laterality of the atrophy alone: the R > L patients tend to have more atrophy extending across the OFC-ATL complex, whilst the anomic-language features in the presentation of the L > R patients may overshadow and/or lead to under-reporting of the accompanying behaviour change. It is also possible that the pronounced language deficits in these patients accelerates their social isolation and thus reduces the opportunities to detect behavioural changes.

### Comparative neurology and other patient groups

Given that semantic dementia always develops some degree of bilateral ATL atrophy and extension to the OFC, findings from other patient groups and comparative neurology provide potentially important insights into the separate roles of each ATL in supporting semantics and social behaviour. Classic comparative neurological studies demonstrated how bilateral, rather than unilateral, surgical removal of the ATLs causes severe chronic behaviour changes and associative agnosia in non-human primates.^62,63^ Following bilateral ATL resection, the monkeys were no longer frightened of guards or predators, were unable to recognize other objects visually (e.g. distinguish between edible and non-edible objects) and no longer recognized the calls of conspecifics or made calls to them.^62^ This syndrome was also seen in a subsequent, thankfully rare, human neurosurgery case.^64^ This combination of symptoms is clearly reminiscent of at least some of the semantic and behavioural impairments observed in semantic dementia.^65^ Indeed, in their seminal papers, Klüver and Bucy^62^ noted the similarities between the resected monkeys and the FTD patients described by Arnold Pick.

In both humans and non-human primates, unilateral ATL resection has a much milder effect than the bilateral ATL atrophy that causes increasingly severe semantic and behaviour impairments in semantic dementia.^62-64,66-68^ In contrast to the striking social and semantic deficits in semantic dementia, people with late-onset TLE who have undergone *en bloc* unilateral ATL resection display mild semantic impairments, which are detected only when more sensitive measures are used.^66-68^ Furthermore, these explorations of unilateral damage have found little evidence for a specialized function of the right ATL in social processing: TLE patients with left or right ATL resection show not only mild but equivalent degradation of person semantic knowledge and emotion recognition, and even when formally assessed, no evidence of altered social behaviours like those observed in bvFTD or semantic dementia.^68^ Of course, data from patients with chronic epilepsy need to be interpreted with some caution given the possibility of cognitive functions being shifted out of seizure centres. Direct cortical grid electrode explorations (stimulation and electrocorticography), however, indicate that the left and right ATLs remain as primary semantic regions even in patients whose epilepsy requires ATL resection, and furthermore that the semantic ventral-ATL ‘hot-spot’ for the patients is identical to the area of maximal fMRI semantic-task activation in healthy participants.^69,70^

### Evidence from neuroimaging and neurostimulation in healthy participants

Functional imaging and neurostimulation methods in healthy participants provide information about the role of different ATL subregions in supporting social-semantic knowledge, providing important extensions to the patient data (reviewed by Pexman *et al*.^71^). Contemporary fMRI studies that have used distortion-corrected or distortion-limiting techniques to enhance signal from the ventral ATLs have demonstrated that semantic processing engages the ATLs bilaterally.^72-74^ Bilateral ATL activation is observed for all types of concepts, including social concepts.^72,75^ This finding is further supported by transcranial magnetic stimulation studies, in which stimulation to either left or right ATL causes a transient disruption of semantic processing in healthy participants.^76,77^

The spatial resolution offered by the recent distortion-corrected fMRI studies has provided important new insights about the roles of different ATL subregions. First, both social-semantic and matched non-social semantic stimuli elicit strong bilateral activation in the ventral ATL, where activation has been observed in numerous other semantic imaging studies.^78,79^ This overlapping activation suggests that social concepts are supported by the same multimodal ventrolateral ATL semantic hub as general semantic memory. A meta-analysis of 97 fMRI studies found bilateral ATL activation for all types of concepts, although there was a left-hemisphere bias for tasks that required either word retrieval or used written words as inputs.^80^ Although a right ATL specialization for social processing has been proposed based on the FTD literature, the meta-analysis found no evidence for hemispheric specialization for social concepts, but bilateral ATL activation for both social and non-social semantic tasks.^80^ Consequently, the fMRI findings in healthy participants support a role for the bilateral ATL in representing all types of semantic memory, including social-semantic knowledge.

Second, moving beyond the ventral ATL region (the subregion affected most strongly by signal drop-out and distortions in standard, single-shot echo-planar imaging), initial fMRI investigations reported activation in the left and right anterior superior temporal gyrus/temporal pole when participants made semantic judgements about abstract social concepts.^27,81^ Furthermore, transcranial magnetic stimulation over these left or right superior ATL areas generates a transient impairment/slowing of social conceptual decisions, which is both cognitively-selective (no slowing of difficulty-matched non-semantic number magnitude judgements) and anatomically-selective (only after ATL but not in anatomical control sites), highlighting the role of superior ATL regions (both left and right) in supporting social conceptual knowledge.^82^ This finding aligns with more recent distortion-corrected fMRI investigations, in which (i) there was more selective activation in the anterior superior temporal gyrus/temporal pole (bilaterally) for social over other types of concepts, but (ii) this anterior superior temporal gyrus/temporal pole activation was weaker than the core ventral ATL activation observed for all types of concepts, including social.^78,79^ The reason for this additional, selective activation in superior temporopolar cortex is not known but may reflect the graded functional organization of the ATLs, where regions outside the core ventrolateral centre-point respond preferentially to different types of concepts depending on their connectivity to other cortical regions.^24,83^ The temporal poles and superior ATL are connected with limbic regions via the uncinate fasciculus, whence emotional valence inputs may be important for the formation of socially relevant concepts.^84-87^ To summarize, there is no strong fMRI or repetitive transcranial magnetic stimulation (rTMS) evidence for a left versus right ATL difference for social concepts, but rather a strong bilateral multimodal ventral ATL response to all types of concepts, with category-selective gradations ‘within’, rather than ‘between’, each ATL.^79,83^

A parallel fMRI literature implicates the ATLs in other aspects of social processing, such as theory of mind/mentalizing, empathy and moral reasoning.^20,88,89^ Theory of mind tasks, alongside social and non-social semantic processing tasks, generate overlapping activation in the dorsal or ventral ATLs.^23,81^ This common activation for theory of mind and semantic processing may reflect a shared and core role of the bilateral ATLs in generalized semantic representation.^23,90^

## The prefrontal cortex: social control

Social-semantic memory alone is not sufficient to support appropriate social behaviour. The knowledge must also be controlled so that it is applied efficiently and used flexibly across different situations and contexts. This is crucial for the generation, implementation or inhibition of adaptive social behaviours across changing social scenarios.^20^ Prefrontal regions such as the orbitofrontal, lateral and medial prefrontal cortex have important roles in representing the value of objects and actions, regulating and inhibiting behaviour.^18,91-93^ Accordingly, we propose that the ‘social control’ component of the model is mediated by prefrontal regions and that it interacts with ATL-mediated social-semantic knowledge to shape social behaviour.

### Behavioural-variant frontotemporal dementia

Although people with bvFTD present with abnormal social behaviours, they do not appear to have the same degree of loss of social-semantic knowledge as in semantic dementia.^94^ Their deficits seem to relate primarily to difficulties in using this knowledge appropriately and flexibly (although social-semantic knowledge may be affected as atrophy spreads into the bilateral ATL^95^). For example, even where there is preserved understanding of abstract social concepts, people with bvFTD are less able to utilize this knowledge to predict long-term consequences of social behaviours and select or decide between alternative actions.^94^ This speaks to the computations of action values or outcomes.^96,97^ In the crying child example above, the decision to comfort the crying child not only depends on an accurate understanding of the meaning of tears but also the positive value of comforting one’s grandchild versus the potential negative consequences of intimacy with other children.

People with bvFTD are less able to adjust the physical space given to a stranger in comparison to a family member, suggesting an inability to control social behaviour in response to changing social contexts.^98^ More broadly, people with bvFTD show cognitive inflexibility^7^ in daily settings and in more formal assessments, such as set-switching^99^ or attentional shift paradigms with reversal of stimulus-reward associations, which are especially challenging.^100^ Another route to inappropriate social behaviours is an impairment of behaviour inhibition, for example where it would be far better not to make a habitual response or react to an affordance.^4,101,102^ The inhibition of prepotent responses (e.g. NoGo paradigms) and inhibition of actions after initiation (e.g. Stop-signal paradigms) are both affected in bvFTD.^4,103,104^

The impairment of these three processes—value-based decision-making, flexibility and inhibition of responses—contributes to poor control of social behaviour. These three processes are each strongly associated with the prefrontal cortex, including its structural and neurochemical integrity. BvFTD typically affects the OFC, medial prefrontal cortex and lateral prefrontal cortex, particularly the ventrolateral prefrontal cortex (Fig. 1B).^105-108^ The OFC is a site of early severe atrophy in bvFTD and has classically been associated with personality and behavioural changes.^18,109^ Some types of apathy, disinhibition and failures in social norm compliance have all been attributed to atrophy or hypometabolism in the OFC.^110-112^

The OFC and medial prefrontal cortex represent reward values of different objects and are important for flexibly controlling behaviour based on changing reward contingencies.^113-116^ Intriguingly, these regions enable estimation of counterfactual value, i.e. relative values of actions or events that are not actually experienced. The loss of value-based decisions following atrophy of the medial prefrontal cortex provides a potential link between socially inappropriate behaviour and the loss of goal-directed behaviour underlying the apathy observed in bvFTD and related disorders.^3,117,118^

Lateral prefrontal atrophy is associated with impaired executive function, which is the set of processes that control cognition, e.g. in working memory, attentional selection, planning and inhibition.^119,120^ Damage to lateral prefrontal cortex also impairs ‘semantic control’: the ability to manipulate and guide semantic knowledge, despite preserved semantic representations *per se.*^24,121-124^ People with post-stroke semantic aphasia following lateral prefrontal lesions have problems with controlling and regulating semantic knowledge,^24,121-125^ including semantic tasks involving emotion and abstract concepts.^126^ In formal meta-analyses of healthy participant fMRI studies, these same semantic control regions are engaged by social cognitive tasks.^20^ Some of the inappropriate social behaviours in bvFTD might therefore be driven partially by disordered social control—the failure of executive processes to guide and control social-semantic knowledge.^20,21,94,127,128^ However, circumscribed lesions to the dorsolateral prefrontal cortex do not cause the severe social disturbances associated with orbitofrontal/ventromedial damage,^129-132^ and semantic aphasia patients with prefrontal damage, including ventrolateral prefrontal, insula and basal ganglia, do not present with the social behaviour disturbances (e.g. apathy, disinhibition) observed in bvFTD. This suggests that OFC and medial PFC are the primary prefrontal regions that underpin controlled social behaviour (although areas beyond the frontal cortex as well as inter-regional white-matter connections might also be important: see later).

The neurocognitive mechanism of social control deficits in bvFTD could be conceptualized in terms of abnormal predictive coding in the brain.^133^ Under the predictive coding framework, the brain uses Bayesian inference to update beliefs about the causes of sensory inputs, and employs such beliefs to predict future sensory inputs.^134^ Impaired behaviour would result from a lack of precision in these beliefs or predictions, with a failure to adapt behaviour appropriately to experience or context.^133^ For example, apathy would result from reduced precision in the predicted consequences of actions, leading to diminished goal-directed behaviour.^133,135^ Impulsive behaviours would follow from reduced precision in the amount of information sampled before a decision is made.^136^ The degradation of conceptual knowledge, including social context, would by analogy impair initiation or selection of socially appropriate actions. This may lead to behaviours that are superficially considered ‘disinhibited’, even without a failure of representational inhibition or action inhibition *per se*.

A powerful feature of the predictive coding hypothesis is that it provides a unified explanation for the co-existence of apparently antithetical symptoms such as apathy and impulsivity, or social apathy and social disinhibition, in the same patient.^3,137^ The known social reward deficits associated with orbitofrontal/anterior cingulate cortex damage may exacerbate the problem, with imprecise predictions of socially relevant informational inputs. For example, inappropriate social behaviours in bvFTD such as social norm violations would result from slow prediction updating in response to important social cues (e.g. an angry or fearful response). The failure of precision is distinct from the ability to compute the expected value of actions (rewards or punishments).^138^

### Comparative neurology and other patient groups

Beyond the FTD literature, evidence for the role of the OFC in social behaviour comes from studies of other patient groups. Damage to the OFC due to traumatic brain injury, aneurysm or stroke causes impairments in social behaviour, aligning with FTD.^139-141^ A famous example is Phineas Gage, who suffered focal OFC damage in an accident in which a tamping iron was driven through his skull.^142,143^ In the acute phase after the accident, Gage displayed changes in behaviour and personality, with socially inappropriate behaviours, despite preserved general intelligence.^143^

Despite the social impairments, focal OFC damage does not seem to disrupt social-semantic knowledge. Such patients display intact semantic knowledge of social norms and conventions.^140,144,145^ OFC damage also impairs performance on reversal learning tasks, which requires participants to adapt flexibly and change their behaviour in response to changing reward contingencies, especially negative feedback.^146^ This deficit occurs despite patients understanding the rules of the task. Reversal performance correlates with behavioural disinhibition after OFC-lesions.^146^ Consequently, it appears that focal OFC damage causes impairments in being able to control behaviour flexibly and respond appropriately to rewards or punishments. Reversal learning deficits have also been demonstrated in OFC-resected monkeys, who perseverate and continue to respond to stimuli which are no longer rewarding.^147^ OFC damage in monkeys causes diminished fear responses to predatory stimuli,^148^ a phenomenon also seen in the classical Klüver-Bucy syndrome,^62^ highlighting how the same impaired behaviour can result from either bilateral ATL or OFC lesions, reflecting damage to representation or control, respectively.

Beyond the OFC, focal lesions to the anterior cingulate cortex (ACC) impair social behaviour in both humans and non-human primates, in line with its role in supporting the control of social behaviour.^129,149^ Apathy has been attributed to lesions in the medial prefrontal cortex in humans, further highlighting the ACC’s role in regulating goal-directed behaviour.^130^

### Evidence from healthy participants

Increased fMRI activation of the OFC is found in response to a wide range of rewarding stimuli,^150-154^ and when healthy participants view violations of social norms.^155,156^ The OFC is also engaged when participants are required to alter behaviour based on changing social reward contingencies.^150,157^ Although fMRI can only provide correlational data, these studies complement the lesion studies described above, highlighting the importance of the region in guiding controlled behaviour.

As with the OFC, the ACC is important for representing value and reward-based decision-making and is thought to support action-outcome learning.^158^ Functional imaging studies in healthy participants have found that the ACC is engaged when reward-related information is processed.^158^ There is evidence that a subregion of the ACC, the ACC gyrus, codes the value of others’ actions, thus computing social predictions necessary for prosocial behaviour.^138,159-163^

## Other proposals for behavioural changes in frontotemporal dementia

The model of controlled social-semantic cognition provides a unifying framework to understand social and semantic impairments across the variations of FTD, and parallel findings in other patient groups, comparative neurology and healthy participants. It aligns with other proposals that have focused on a particular patient group, brain area or process. We briefly consider three important proposals below and note their relationship to the broader CS-SC framework. A recurring theme across all models is the interaction of prefrontal and temporal regions in supporting social behaviour, though the exact areas and their proposed functions vary.

### Social Context Network Model

The Social Context Network Model (SCNM) proposes that the social deficits in bvFTD result from an inability to use context to guide behaviour, following damage to a network of brain regions including the prefrontal cortex, insula and medial temporal lobes.^164,165^ According to the SCNM, prefrontal cortex is critical for the generation and updating of context-driven predictions and interacts with medial temporal regions to support learning of contextual associations. The insula acts as a convergence hub for internal and external signals to produce global feeling states. Thus, as per the CS-SC framework, the SCNM emphasizes the importance of prefrontal regions and their interaction with other regions in supporting appropriate social behaviour. With its focus on bvFTD and prefrontal regions, the SCNM is silent on the behaviour changes in semantic dementia, the ATL regions and social-semantic knowledge.

### Salience and semantic appraisal networks

In addition to individual brain regions, a recent proposal has considered FTD behaviour changes in terms of damage to large-scale brain networks.^96,166^ The salience network has hubs in the anterior insula and ACC, areas that are systematically affected in bvFTD.^109,167,168^ The salience network is thought to support attention to and engagement with salient stimuli. Damage to this network would result in a failure to recognize and react to important/salient social signals.^96,169^ More specifically, within this network, the anterior insula might integrate interoceptive cues to generate feeling states, and the ACC recruit executive processes to guide behaviour in response to salient stimuli.^167^

Second, the semantic appraisal network^106^ is particularly affected in people with semantic dementia and also in some people with bvFTD.^170^ The semantic appraisal network has a core hub in the ATLs, with nodes in limbic regions including the amygdala and OFC.^29,167^ In this proposal, the ATLs are considered to represent social-semantic knowledge, which is tagged with hedonic value represented in the ventromedial/orbitofrontal cortex.^58^ Damage to this network would therefore lead to social concepts being stripped of their meaning and value, leading to impaired social behaviour. It has recently been proposed that a loss of social-semantic knowledge following ATL atrophy might also be a contributing factor to behavioural disinhibition in FTD syndromes.^128^ These network proposals are closely aligned with the CS-SC framework and the broader theory of controlled semantic cognition in which the ATL hub interacts with multiple ‘spoke’ regions to generate coherent concepts, and this semantic network interfaces with areas related to executive function in order to generate controlled, context/time-appropriate behaviours.^21,24,35^ Under the salience and semantic appraisal networks approach, the orbitofrontal area (like the insula) is considered to contribute a specific source of information (hedonic/valence value) to the ATL semantic-hub rather than support a more executive, evaluation computation.

### Event-feature-emotion complexes

This framework proposes that the ATLs and prefrontal cortex each store distinct aspects of social knowledge, which interact to support flexible social behaviours.^171^ In this framework, context-independent semantic knowledge of social concepts is stored in the superior aspects of the ATLs, whereas context-dependent event sequences (‘scripts’) are stored in the prefrontal cortex.^171,172^ Subdivisions of the prefrontal cortex are proposed, such that the ventromedial prefrontal cortex stores socially relevant scripts, and the frontopolar cortex stores long-term event sequences required for anticipation of long-term future consequences of behaviours.^94,172,173^ Event-feature-emotion complexes emerge from the integration of ATL-based context-independent knowledge, prefrontal-based context-dependent knowledge, and central motive states represented in paralimbic and limbic regions.^171^

As per the CS-SC, this model postulates dissociable yet interacting roles of the ATL and prefrontal regions in supporting social behaviour. Both models suggest that context-independent semantic knowledge is supported by the ATLs.^171^ A key difference is that the event-feature-emotion complex framework implicates the prefrontal cortex as a long-term memory store for social events, whereas the CS-SC framework suggests the prefrontal cortex has a control function in guiding and regulating social-semantic knowledge.

## Other important brain areas and connectivity

In this review, we have primarily considered the roles of the ATLs and the prefrontal cortex in supporting social behaviour. However, there are other brain regions and white-matter connections, beyond the frontotemporal complex, which are affected by FTD. Additional research is needed to explore if and how these brain regions also contribute to the various social deficits in FTD. In line with the convergent approach advocated in this review, it also seems important to garner data on each of these possible contributory brain areas through parallel explorations in complementary non-FTD patient groups, comparative neurology and healthy participants. Such studies will help to delineate the specific contributions of each additional area and also guard against false positive localization of function due to the multiple areas of correlated atrophy in FTD.

The insula has attracted significant interest for multiple reasons: it is consistently atrophied in FTD,^174^ and is a key node within the ‘salience network’ and thus a potential crucial nexus when considering FTD as a network-aligned disease process.^106^ Early *in vivo* human tractography studies showed that the anterior insula is part of a white-matter loop with the temporal pole and orbitofrontal cortex, whereas dorsal-posterior insula connects more into language-related areas.^175,176^ As noted earlier, in the ‘Other proposals for behavioural changes in FTD’ section, there has been increasing interest in the role of interoception in socio-emotional processing and the potential importance of the insula in FTD.^165,167,177^ Future work is required to explore how the role of interoceptive processes is accommodated within the CS-SC framework. One preliminary hypothesis, consistent with the network theories described above in the ‘Salience and semantic appraisal networks’ section, is that the insula represents an ‘interoceptive spoke’ which feeds into the ATL semantic system. Careful examination of different aspects of behavioural change in FTD has associated inappropriate trust/approaching behaviour with atrophy of the insula-amygdala ‘aversive’ network,^178^ whilst multivariate imaging analysis has indicated that sarcasm and emotion recognition deficits may be dependent on the entire insula-OFC-amygdala-temporal pole network.^179^ Extending these ideas a little further, prefrontal-basal ganglia circuits have been associated with different aspects of apathy. More specifically, it has been suggested that ‘emotional–affective’ apathy may be related to damage within the medial prefrontal-orbitofrontal-ventral striatum network.^118^ One recent large-scale FTD investigation^180^ found that apathy and anhedonia were significantly increased in both bvFTD and semantic dementia, and were behaviourally correlated. Both were associated with atrophy of the orbito-ventromedial-polar frontal areas, while correlations were also found for anhedonia with the insula and putamen.

The CS-SC framework implicates a network of individual yet interacting brain areas in supporting social behaviours. Accordingly, it is likely that the white-matter connections between the key areas are critical too. The uncinate fasciculus, anterior commissure and other parts of the extreme capsule complex provide the major white-matter connections between the ATLs, the OFC, prefrontal regions and other potentially important additional areas such as the insula.^175,176^ These connections will provide the basis for the interaction of social control, social-semantic representations and other critical inputs.^21,181^ Indeed a recent study^95^ associated reduced fractional anisotropy in the uncinate fasciculus with bvFTD patients’ highly irregular emotional reactions to personal high-conflict moral dilemmas (even though their adjudication between moral decisions was the same as control participants and patients with Alzheimer’s disease).

## Directions for future research and clinical implications

We propose four priority areas for future research and clinical application.

### Varieties of social concepts?

One important avenue for further exploration is to test the contributions of ventral and dorsal ATL regions to social-semantic conceptual processing; and to determine, more broadly, how different types of social concepts are represented in the brain. For example, are they ‘special’ and distinct from other types of general (i.e. non-social) concepts as implied by earlier research^19,27,182^ or an integral part of the broader conceptual system?^21,24,28^ More broadly, research on social behaviour and the underlying representations is complicated by the fact that many patient and healthy participant studies investigate different individual ‘social’ concepts. These ‘social’ concepts span very diverse types of semantic representation (that are likely to have varying reliance on multiple brain regions), from very concrete entities such as people, through emotions, to more abstract behaviours and social traits. Consequently, it becomes less clear what crucial characteristic(s) make a concept ‘social’^71,183^ or whether, like Wittgenstein’s famous ‘game’ concept problem,^184^ there is no single defining feature shared by all social concepts.^26^

### Frontotemporal interactions

As noted earlier, all proposals highlight the importance of distinct functions/representations in prefrontal and temporal areas. Thus, a primary next step is to understand their interaction at a functional-mechanistic level. The polar, medial and superior aspects of the ATLs are strongly connected with the orbitofrontal and ventromedial cortex, with this connectivity taking up the bulk of the uncinate fasciculus.^84,85,87^ Thus, it is important to understand the functional contributions that these structural connections support.^185^ For example, how does ATL-based social-semantic knowledge interact with OFC-based value computations in humans?^97^ When deciding to perform a behaviour, the value of any object is highly dependent on its meaning. For example, if someone is hungry, the value of a round object will be higher if it is an apple as opposed to a cricket ball. It would then logically follow that ATL-based semantics would be a key input to OFC-based value computations; and in return, the OFC ‘valence/value’ information (akin to any of the other sensory-motor and verbal sources of information codes across different association cortices) interacts with the ATL semantic hub to support concepts where valence/value is important.^96,97^

### Transdiagnostic approaches to assessment and clinical research

Whilst the CS-SC and other proposals posit discrete functions/representations to prefrontal versus ATL regions, bvFTD and semantic dementia patients do not divide absolutely and selectively along the same anatomical division. Notwithstanding distinct clinicopathological correlations with underlying molecular aetiologies, there are patient exemplars of classical bvFTD and semantic dementia representing different phenotypic points along a frontotemporal atrophy continuum, with many other ‘mixed’ FTD patients being intermediate. Accordingly, group-level comparisons provide important general clues about broad distinctions within FTD but are not optimal for understanding (i) the distinct functions of prefrontal versus ATL regions; and (ii) systematic variations and shared symptoms that span FTD subtypes. These features can be revealed by adopting a transdiagnostic approach and multidimensional analytics^16,17,33^ and the results supplemented by convergent information from other patient groups and healthy participants.

### Clinical assessments, diagnosis and management pathways

Inspired by the CS-SC framework, the development of new neuropsychological tests able to distinguish between degraded social-semantic representations versus social control problems would provide strong clues about the neural and cognitive bases driving a patient’s behaviour change. In doing so, it may be possible to improve the delineation between semantic and behavioural variants of FTD, as well as understand the range and severity of problems faced by the many FTD patients with a mixed neurocognitive profile. Such group comparisons combined with transdiagnostic explorations could help lead us towards (i) better understanding of the underlying anatomical changes, pathology and genetic factors; and (ii) tailoring of both behavioural management and pharmacological interventions for the different types of deficits in social cognition.
